# Predicting Cell Cycle Regulated Genes by Causal Interactions

**DOI:** 10.1371/journal.pone.0006633

**Published:** 2009-08-18

**Authors:** Frank Emmert-Streib, Matthias Dehmer

**Affiliations:** 1 Computational Biology and Machine Learning, Center for Cancer Research and Cell Biology, School of Biomedical Sciences, Queen's University Belfast, Belfast, United Kingdom; 2 Institute of Discrete Mathematics and Geometry, Vienna University of Technology, Vienna, Austria; University of Manchester, United Kingdom

## Abstract

The fundamental difference between classic and modern biology is that technological innovations allow to generate high-throughput data to get insights into molecular interactions on a genomic scale. These high-throughput data can be used to infer gene networks, e.g., the transcriptional regulatory or signaling network, representing a blue print of the current dynamical state of the cellular system. However, gene networks do not provide direct answers to biological questions, instead, they need to be analyzed to reveal functional information of molecular working mechanisms. In this paper we propose a new approach to analyze the transcriptional regulatory network of yeast to predict cell cycle regulated genes. The novelty of our approach is that, in contrast to all other approaches aiming to predict cell cycle regulated genes, we do not use time series data but base our analysis on the prior information of causal interactions among genes. The major purpose of the present paper is to predict cell cycle regulated genes in *S. cerevisiae*. Our analysis is based on the transcriptional regulatory network, representing causal interactions between genes, and a list of known periodic genes. No further data are used. Our approach utilizes the causal membership of genes and the hierarchical organization of the transcriptional regulatory network leading to two groups of periodic genes with a well defined direction of information flow. We predict genes as periodic if they appear on unique shortest paths connecting two periodic genes from different hierarchy levels. Our results demonstrate that a classical problem as the prediction of cell cycle regulated genes can be seen in a new light if the concept of a causal membership of a gene is applied consequently. This also shows that there is a wealth of information buried in the transcriptional regulatory network whose unraveling may require more elaborate concepts than it might seem at first.

## Introduction

In recent years large parts of biology, especially molecular and cell biology, have been transformed gradually into fields driven by technological progress. This has been initiated by the development of high-throughput techniques like, e.g., DNA microarray or yeast two-hybrid. These new experimental technologies allow now to measure on a genomic scale molecular biological entities and, hence, an analysis on a systems level [1], [2], [3], [4]. Due to the fact that a functional understanding of a living cell can only be achieved by studying interactions among genes or products thereof network based analysis methods have attracted much attention [5], [6], [7], [8]. For this reason we are now facing the difficulty to analyze gene networks, e.g., metabolic, signaling or the transcriptional regulatory network [9], [10], [4], [11] to extract from them sensible biological information.

In the present paper the major purpose is to use the transcriptional regulatory network of yeast to predict cell cycle regulated genes of *Saccharomyces cerevisiae* by using a novel approach. For predicting cell cycle-regulated genes, which are also called *periodic genes* [12], we use the transcriptional regulatory network of yeast and a list of known genes to be periodically expressed during the cell cycle. No other data are used. This means explicitly that we do not use time series data from, e.g., DNA microarray experiments that would allow to test statistically for periodic behavior or appearance of genes. We want to emphasize that our approach is fundamentally different to all other approaches we are aware of predicting periodically expressed genes for the cell cycle of yeast [13], [14], [15], [16], [17], [18] because all other approaches are based on time series data. This may seem counter intuitive at first sight, however, the seeming contradiction is resolved quickly. First, we want to repeat that we and all other studies are aiming to detect genes that are cell cycle regulated. That means genes that *belong to* or *participate in* a certain biological process namely the cell cycle. However, from a biological point of view this means we are searching for genes that have a biological function that is important for the biological process cell cycle. Hence, in statistical terms we are searching for genes that are *causally* connected to the cell cycle. This brief explanation makes clear that there is no need to quantify or qualify further entities including, e.g., the *periodicity* of genes regarding the shape of their signal, to enhance our definition. The causal membership of a gene in the biological process *cell cycle* is sufficient to study this problem provided we take information into account regarding the causal interaction paths connecting periodic genes. For this reason we use the transcriptional regulatory network.

In a previous work we used already the concept of a *causal membership* of a gene to study the organizational principle of the cell cycle of yeast [19]. There we analyzed a subnetwork of the transcriptional regulatory network and could demonstrate that the obtained subnetwork is statistically significant with respect to several properties, e.g., the number of periodic genes reachable from the strongly connected component (SCC). Further, we hypothesized that this subnetwork may act as a pacemaker of the cell cycle itself because the implied hierarchy between periodic genes is directed from periodic genes in the SCC to genes outside and only genes in the SCC can exhibit truly periodic behavior due to the cyclicity of the SCC. In the present paper we do not focus on the network topology or study structural properties thereof but utilize its topology to make functional predictions regarding genes that are cell cycle regulated. Our prediction will utilize the concept of a *causal membership* of a gene.

The paper is organized as follows. In the next section we introduce our method and describe the data we use for our analysis. In the ‘results’ section we presents numerical results and this article finishes with conclusions.

## Methods

### Data

For our analysis in the following we use the transcriptional regulatory network (TRN) of yeast [20], [21]. From this network we extract the weakly connected component (WCC) which consists of 3357 genes and 7230 interactions. The weakly connected component of a network is defined as the subnetwork that connects every pair of nodes by at least one directed path [22]. In contrast, the strongly connected component (SCC) is defined as subnetwork that connects each pair of genes in both directions that means for each pair of genes A and B there exists a directed path from gene A to gene B but also a directed path from gene B to gene A. The TRN consists of two strongly connected components. One consists of 36 and the other of just 2 genes. When we speak in the following about the SCC of the TRN we speak always about the larger subnetwork also called the giant strongly connected component [23]. The strongly connected component is part of the weakly connected component, 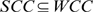. We use a list of Zhao et al. as reference for periodic genes [18]. In this list they categorized 260 genes as periodic. However, only 179 periodic genes are in the WCC we use for our analysis. We restrict our analysis to the WCC because the WCC can be seen as filtered network providing the highest quality subnetwork of the TRN.

### Method

In this paper we use the transcriptional regulatory network (TRN) of yeast that has been assembled from different types of high-throughput data [20], [21] to ensure that the interactions present in the network correspond to real biologically observable interactions (low number of false positive edges) and, hence, to represent a causal interaction structure. We study the structure of this causal network to predict cell cycle regulated genes which are also called periodic genes. Because all other approaches suggested so far to predict periodic genes are based on statistical tests comparing differences in signal shapes of time series data from microarray experiments [13], [14], [15], [24], [16], [17], [18] we first define some terms for clarification. More precisely, we want to emphasize again that for our prediction we use only the TRN of yeast and a list a genes known to be periodic to predict novel periodic genes. We do not use time series data of any kind.

Of central importance for our analysis is the notion of the *causal membership* of a gene introduced in [19].

#### Definition 1 (causal membership)

*The causal membership is an indicator function that indicates if a gene* 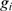 *belongs to a certain biological process.*

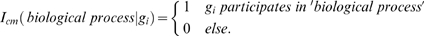



Definition 1 emphasizes the fact that when talking about the biological function of a gene we are interested in the causal involvement of a gene in a certain biological process instead of mere biochemical properties. From this perspective it appears natural that genes participating, e.g., in the biological process cell cycle can be studied with the help of a causal network representing interactions among these genes.

In the following we make the assumption that the transcriptional regulatory network represents all possible causal interactions among genes. No other interactions can occur.

#### Assumption 2

*The transcriptional regulatory network G represents all possible causal interactions among genes.*

It is clear that our assumption is not entirely true because there is also communication among genes involving, e.g., phosphorylation or signaling in general. However, as with all assumptions, we will only know about its quality after we performed the analysis on which the study has been based on. As we demonstrate in the results section, despite the apparent incompleteness of our assumption the transcriptional regulatory network seems to make a very strong contribution.

Our study is motivated by the following hypothesis.

#### Hypothesis 3

*Given a directed causal path, obtained from the transcriptional regulatory network, connecting two genes known to be periodic then all genes on this path are periodic if the following two conditions hold:*

1. *the connecting path is a shortest path.*
2. *there is just one shortest path connecting the periodic genes.*

The reason why we formulated this as a hypothesis rather than a theorem is that we *assumed* that the significant (molecular) interaction path follows the shortest path connecting two genes. Despite the fact that this assumption is frequently made [25], [10], [26] it is not possible to rule out that also non-shortest paths might be used at least under certain conditions. Hence, there is a certain, difficult to quantify, uncertainty attached to this hypothesis. However, a slight modification of the conditions transform Hypothesis 3 into a theorem.

#### Theorem 4

*Given a directed causal path, obtained from the transcriptional regulatory network, connecting two genes known to be periodic then all genes on this path are periodic if there is only one path connecting the periodic genes.*

The condition in Theorem 4 implies that there is just one shortest path namely the path itself.

#### Proof 5

*Because we assume that the transcriptional regulatory network represents all possible causal interactions (assumption 2) and information can only be transmitted via causal interactions there is just one path along which the information can be transmitted between the two periodic genes.*

We want to remark that we do not allow auto-regulations of a genes. From the proof of Theorem 4 we can see that the periodicity of genes does not enter the proof. More precisely, that means it is not necessary to consider the shape of a signal to make statements about the periodic behavior of genes. Instead, a causal membership in the form *participating* in the information transmission between periodic genes defines genes as periodic (for the condition in Theorem 4).

From a practical point of view, however, there is a problem that might limit the use of Theorem 4. The problem is that for a known list of periodic genes of the order 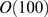 (for example [23]) one needs to study more than 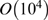 connections between periodic genes. Here the problem is not just computational but conceptional because it seems unreasonable to assume that, in principle, every gene can communicate with every other gene. It is implausible because it implies a homogeneity among genes. Instead, it is widely assumed that genes and, hence, gene networks, are hierarchically organized [27], [28], [29]. In the following we report a property of the TRN that allows to introduce a two-level hierarchy that in turn not only reduces the computational complexity considerably but also results in a novel conceptual view of the cell cycle.

The transcriptional regulatory network can be partitioned by the presence or absence of cycles connecting genes. In mathematical terms a part of the network that is cyclic is also called a strongly connected component (SCC) [22]. This leads us to the separation of the genes in two classes. The first class consists of genes that belong to the SCC. The genes in the second class do not belong to the SCC. Further the two classes are not equal but the information should flow in one direction namely from 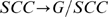. (Here ‘
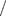
’ is the difference operator giving a new set whose elements are only in *G* but not in *SCC*.) The reason is that only genes in the SCC can establish a periodic behavior, as explained above, while genes in 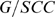 can not. Based on this classification and hierarchy which implies a main direction of information flow among periodic genes, we refine our hypothesis by restricting the set of genes from which we are searching the shortest paths to the SCC.

#### Hypothesis 6

*Given a causal path from a gene in the SCC to a gene in* 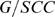 *, obtained from the transcriptional regulatory network, connecting two genes known to be periodic then all genes on this path are periodic if:*

1. *the connecting path is a shortest path.*
2. *there is just one shortest path connecting the periodic genes.*

In the results section we apply Hypothesis 6 to the transcriptional regulatory network of *Saccharomyces cerevisiae*.

## Results

### Subnetwork consisting of periodic genes

**Figure 1:**
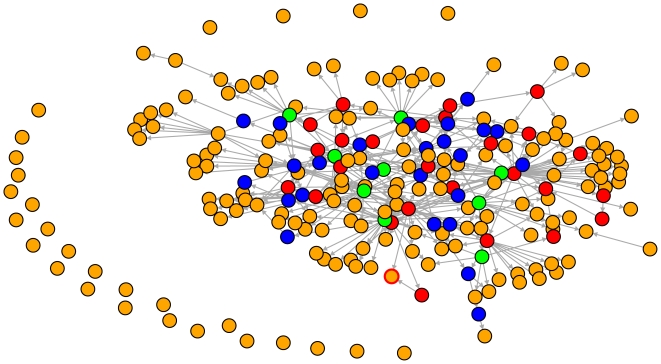
Subnetwork of the TRN of yeast. Shown are 230 genes. Nodes in orange correspond to periodic genes that are not in the SCC (170), green genes are periodic and in the SCC (9), red genes (27) are in the SCC but are not periodic and blue nodes (25) are genes not categorized as periodic according to [18]. The connections shown are shortest paths connecting the periodic genes. All other connections are omitted.

We begin our analysis by showing a subnetwork of the transcriptional regulatory network containing all periodic genes. This network in Fig. 1 was obtained by searching for each periodic gene the shortest paths to all other periodic genes. If a path exists, connecting two periodic genes, all genes on this path are shown in Fig. 1. In Fig. 1 we use a color coding to distinguish genes with different properties. Genes in orange (170) are periodic genes that are not in the SCC, genes in green (9) are periodic and in the SCC, genes in red (27) are in the SCC but are not periodic and blue genes (25) are not periodic and not in the SCC. We want to emphasize that Fig. 1 shows a raw or unorganized version of a subnetwork of the transcriptional regulatory network of yeast. The major purpose of our analysis in the following will be to transform this unorganized network into a representation that can be analyzed sensibly. Before we proceed we want to make some general remarks.

From Fig. 1 there are two things one sees immediately. First, there are many genes that are completely unconnected. Second, the leaf nodes of this subnetwork are periodic genes not in the SCC (orange nodes). If a gene is unconnected (see on the left side in Fig. 1) this means that there exists no path to or from any other periodic gene in the whole transcriptional regulatory network. This means, according to the transcriptional regulatory network we use there is no communication possible between the unconnected periodic genes and all other periodic genes. For this reason, for our analysis in the following these unconnected genes will not be taken into account. The fact that only orange genes and no green ones are leaf nodes (we inspected all green genes - leaf nodes have no out-going edges) indicates an asymmetry. This asymmetry which can also be seen as hierarchy because the leaf nodes are apparently dead end streets regarding information flow (no information can leave towards other periodic genes) is a central part of our hypothesis 6 we raised in the ‘methods’ section. The remainder of the results section is concerned with the organization of the network in Fig. 1 by application of our hypothesis 6.

### Predicting periodic genes

In the previous subsection we showed a subnetwork of the transcriptional regulatory network that was obtained by searching shortest paths between all periodic genes. Now we apply hypothesis 6 and use only parts of this network which is obtained as follows. We search for all periodic genes in the SCC all shortest paths to all other periodic genes. These are apparently less paths because, first, we search only from a subset of all periodic genes and, second, we are no longer interested in paths starting from periodic genes outside the SCC. This results in a network containing only nine non periodic genes that are not part of the SCC (instead of 27 in Fig. 1). Furthermore, this network connects 141 periodic genes which corresponds to almost 80% of all periodic genes in the WCC we are using for our analysis. A statistical analysis has shown that the structure of this network as well as the number of connected periodic genes is unlikely to be observed by chance and, hence, may manifests evolutionary information encoded in the structure of the transcriptional regulatory network [19]. In the following we are focusing on these nine non periodic genes and all other genes they are connected to.

Table 1 shows the length of the shortest paths from all 9 periodic genes in the SCC (first row) to 12 periodic genes (first column) connected via at least one non-periodic gene (blue node). From this table we see that there are only four periodic genes (WSC2, SPH1, EEB1, YLL032C) that can be reached via just one shortest path. All other genes are reachable via multiple shortest paths. For example, MNN1 can be reached from RAP1 and SPT16 via paths both having length 2. For this reason in the second row in Table 1 there are two brackets () indicating that there are two shortest paths to MNN1.

**Table 1:** Length of the shortest paths from all nine periodic genes in the SCC (first row) to periodic genes in 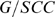 connected via at least one non periodic gene (first column).

	REB1	RAP1	HCM1	YOX1	PHO4	SPT16	ACE2	TOS4	FKH2
WSC2	4	3	4	3	12	3	4	(2)	5
MNN1	3	(2)	3	3	9	(2)	3	5	4
SPH1	3	(2)	10	10	11	9	8	7	9
ERG3	5	4	(2)	3	6	2	3	(2)	4
EEB1	6	5	6	6	12	5	6	5	(4)
YLL032C	5	4	6	7	13	8	9	(3)	6
TAO3	(2)	4	3	5	9	5	6	3	(2)
YLR049C	(2)	4	3	5	9	5	6	3	(2)
PCL7	(2)	(2)	(2)	3	9	4	5	3	(2)
FLC3	(2)	4	3	5	9	5	6	3	(2)
KEX2	(2)	4	3	5	9	5	6	3	(2)
YFL064C	(2)	4	3	5	9	5	6	3	(2)

For example the length of the shortest path from REB1 to WSC2 (first line) is 4. The number in brackets indicates the length of the minimal shortest paths.

**Figure 2:**
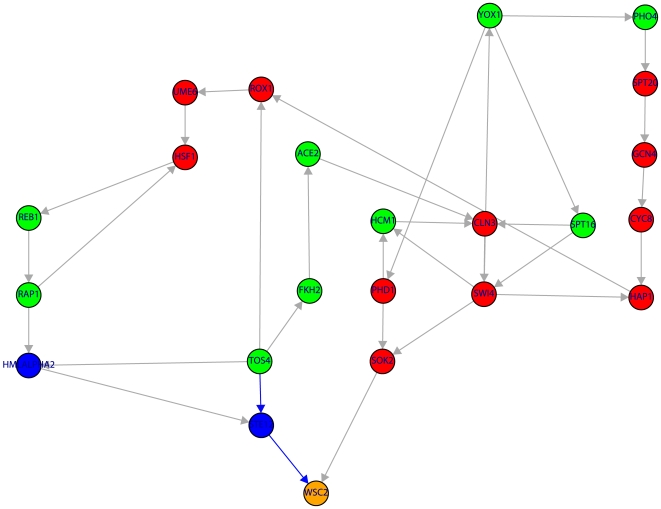
Subnetwork of the TRN consisting of 23 genes (color code as in Fig. 1). The shown subnetwork complements the results in table 1 by providing detailed information about the genes involved in the shortest paths. Blue edges indicate the shortest path from TOS4→STE12→WSC2.

**Figure 3:**
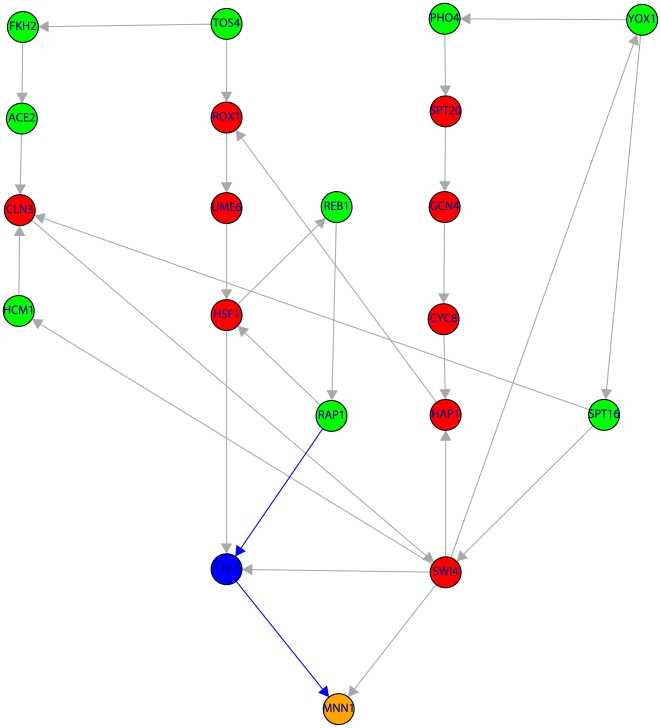
Subnetwork of the TRN consisting of 20 genes (color code as in Fig. 1). The shown subnetwork complements the results in table 1 by providing detailed information about the genes involved in the shortest paths. Blue edges indicate the shortest path connecting RAP1→TYE7→MNN1.

**Figure 4:**
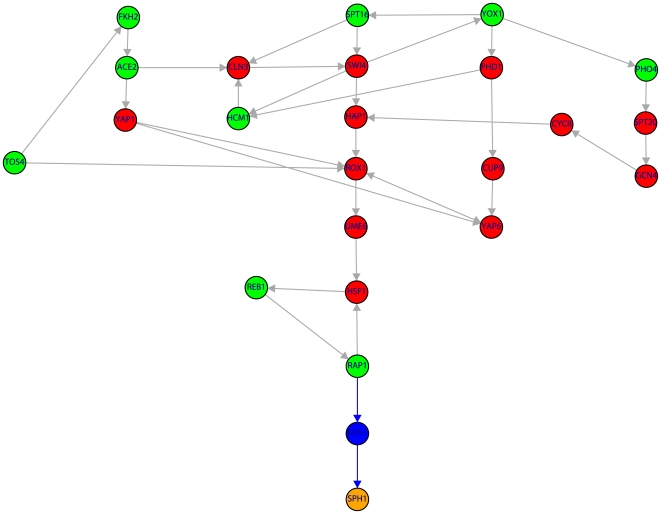
Subnetwork of the TRN consisting of 24 genes (color code as in Fig. 1). The shown subnetwork complements the results in table 1 by providing detailed information about the genes involved in the shortest paths. Blue edges indicate the shortest path connecting RAP1→RPH1→SPH1.

**Figure 5:**
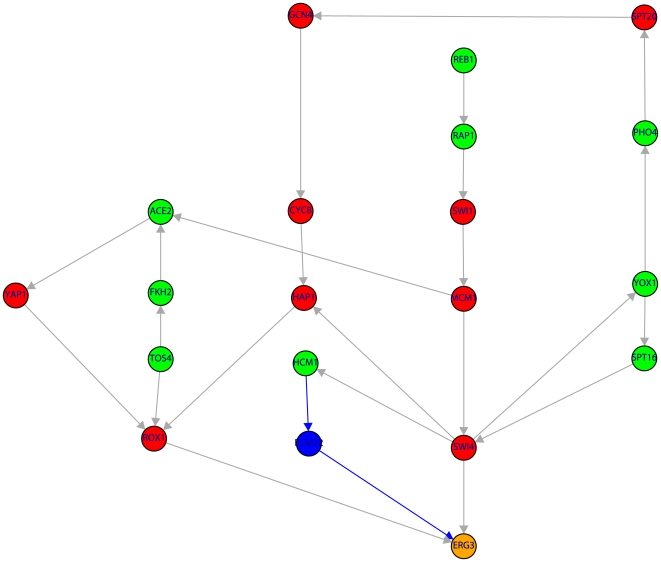
Subnetwork of the TRN consisting of 20 genes (color code as in Fig. 1). The shown subnetwork complements the results in table 1 by providing detailed information about the genes involved in the shortest paths. Blue edges indicate the shortest path connecting HCM1→ECM22→ERG3.

**Figure 6:**
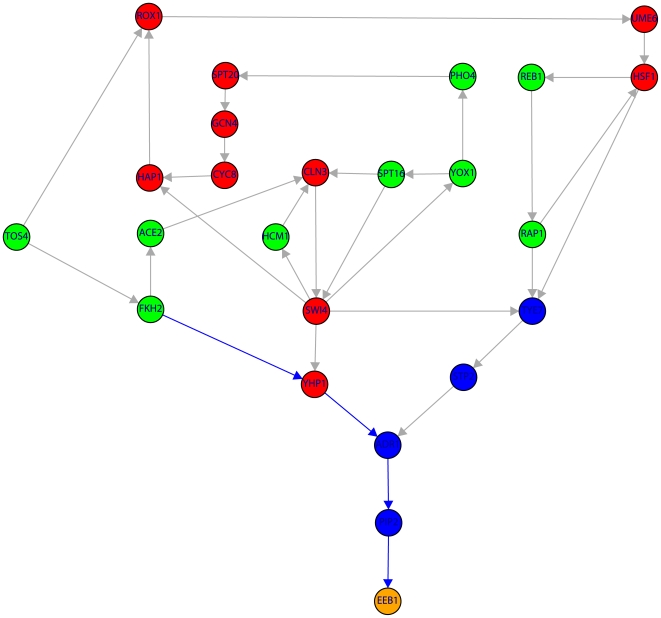
Subnetwork of the TRN of yeast containing 24 genes. Color code of the nodes is as in Fig. 1. The shown subnetwork complements the results in table 1 by providing detailed information about the genes involved in the shortest paths. Blue edges indicate the shortest path connecting FKH2→YHP1→ADR1→PIP2→EEB1.

**Figure 7:**
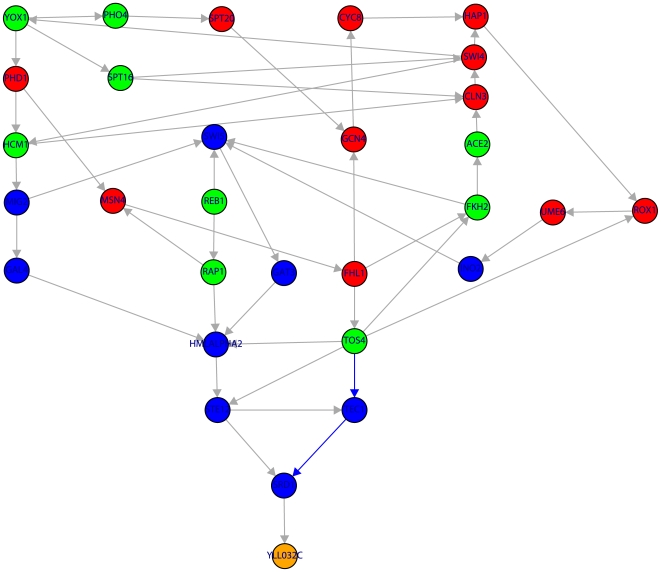
Subnetwork of the TRN of yeast containing 30 genes. Color code of the nodes is as in Fig. 1. The shown subnetwork complements the results in table 1 by providing detailed information about the genes involved in the shortest paths. Blue edges indicate the shortest path connecting TOS4→TEC1→SRD1→YLL032C.

**Figure 8:**
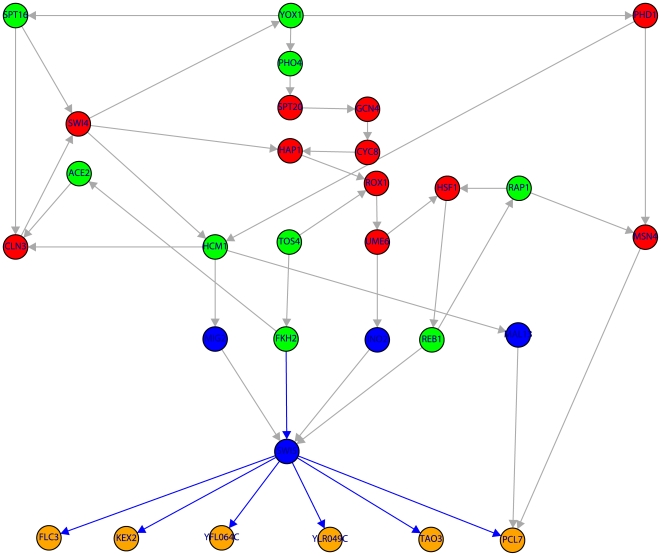
Subnetwork of the TRN of yeast containing 30 genes. Color code of the nodes is as in Fig. 1. The shown subnetwork complements the results in table 1 by providing detailed information about the genes involved in the shortest paths. The blue edges indicate the shortest path connecting FKH2→SWI5→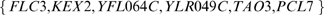.

Figure 2 to 8 visualize these results by showing the subnetwork of the TRN that connects the nine periodic genes in the SCC (green nodes) to the periodic genes (orange nodes) via shortest paths (only the shortest paths are shown). Due to the fact that many of these shortest paths go through non-periodic genes in these figures are also red nodes which correspond to non-periodic genes in the SCC and blue nodes corresponding to non-periodic genes outside the SCC.

In Table 2 we list all nine genes that are candidates to be periodic according to Fig. 2 to 8 (blue nodes on shortest paths). Considering information from the literature we find that Cyclebase [30] declares TEC1 and SWI5 as periodic genes that appear not in the list of Zhao et al. [18]. These genes are also ranked low by two further studies [12] and [31]. By including this information in our analysis this explains the connectivity from FKH2 to 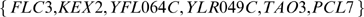 shown in Fig. 8 completely because now we found a shortest path consisting of only genes to be known to be periodic. This leaves us with seven candidate genes to be periodic.

**Table 2:** Nine candidate genes (first column) to be periodic.

gene	Johnansson et al.	de Lichtenberg et al.	Cyclebase
STE12	2888	2421	3885
TYE7	3131	753	1618
RPH1	1989	2877	4022
ECM22	3997	2710	3636
ADR1	3455	4029	2238
PIP2	5650	4762	1277
TEC1	239	104	319 (per)
SRD1	5871	2247	2882
SWI5	109	79	124 (per)

Genes declared to be periodic by Cyclebase are indicated by (per). The numbers in the second, third and fourth column correspond to the ranking according to Johnansson et al. [31], de Lichtenberg et al. [12] and Cyclebase [30].

For the genes in the SCC that are non-periodic we perform a similar literature search which results are listed in Table 3. Also for these genes we find five genes (CLN3, SWI4, MCM7, YHP1, PHD1) that are declared periodic by Cyclebase [30]. Using this information two further scenarios shown in Fig. 3 and 5 are clarified and demonstrated to be conform with our hypothesis. This implies also that neither TYE7 nor ECM22 needs to be periodic because we found alternative (shortest) paths. We want to make clear that this does not give us information to make the statement that these genes are not periodic. They may be periodic but based on our analysis we can not support this hypothesis because we found alternative (shortest) paths to connect MNN1 (Fig. 3) and ERG3 (Fig. 5) to periodic genes in the SCC.

**Table 3:** All non-periodic genes in the SCC.

gene	Johnansson et al.	de Lichtenberg et al.	Cyclebase
CLN3	344	781	158 (per)
SWI4	149	402	122 (per)
SIN3	3768	4465	5029
CYC8	2213	2738	1375
HAP1	3624	1749	2404
MOT3	2070	2942	1193(4.946E-4, 0.0269)
MCM7(YBR202W)	64	53	70 (per)
ROX1	1409	2094	1027(1.756E-4, 0.0162)
YHP1	147	236	282 (per)
YAP6	1098	2003	2995
HPR1	2892	4927	5712
GCN4	3200	5037	2975
UME6	5478	2132	791 (0.0012, 5.597E-4)
HSF1	2806	3541	2033
CIN5	1364	725	832 (0.0032, 2.298E-4)
GLN3	5190	5979	5580
SOK2	3645	3801	2501
SPT20	4755	5901	5022
SWI1	1388	1675	3459
YAP1	5681	4483	6139
PHD1	690	175	495 (per)
MSN4	3877	1379	1192 (0.0064, 0.0034)
CUP9	5746	3546	1094 (2.794E-4, 0.0201)
FHL1	636	1455	722
BDF1	2836	2949	2728
MCM1	3544	1639	3311
NOT5	4380	6103	4635

The first column gives the gene name, the second and third give the rank of the gene according to Johnansson et al. [31] and de Lichtenberg et al. [12] and the fourth column gives the ranking according to Cyclesbase [30]. In brackets we indicate if a gene is declared periodic (per) or alternatively the p-values (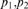) for periodicity (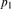) and regulation (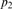).

Considering in addition also genes that have a low p-value for periodicity and regulation (shown in Table 3) according to Cyclebase [30] but without a clear defined peaking point during the cell cycle the number of candidate genes to be periodic can not be further reduced. Hence, there remain only five candidate genes we predict to be periodic (STE12, RPH1, ADR1, PIP2, SRD1) (see Fig. 2, 4, 6 and 7) according to our analysis for which we could not find information from the literature to back up our prediction. All these genes are involved in a single (shortest) path, as demonstrated by table 1, connecting a periodic gene from the SCC to a periodic genes outside.

### Assessing errors

Our analysis presented above rests on the assumption that the used transcriptional regulatory network corresponds to the true (error free) TRN of yeast. Despite the fact that we filtered the TRN using only the WCC this assumption is certainly over-optimistic. For this reason the question arises what influence does the addition or removal of interactions (edges in the network) have on our results. To simplify the analysis we assume in the following either false positive or false negative edges but not both types at the same time. We would like to estimate the probability 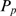 that our prediction is wrong, that means the probability that a non-periodic gene we predict to be periodic is actually non-periodic. Hence, 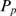 is the false positive probability of a prediction. Because this is for combinatorial reasons intricate we estimate the probability that ‘the non-periodic gene does not need to be periodic’ as approximation for 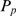. This implies that a new path needs to be either used or established to connect the periodic genes with each other. Different cases are discussed in detail in the following.

For evaluating the effect of false negative edges (addition of edges) we assume that the probability of a false negative edge is *γ* to connect two genes. From our analysis of the subnetwork we obtained (not shown) that there are three principle scenarios we need to distinguish. One, two periodic genes are connected via one non-periodic gene. Two, two periodic genes are connected via two non-periodic genes and, three, there is one non-periodic gene used to connect to more than one periodic gene (the non-periodic gene occurs on multiple shortest paths to periodic genes).

For scenario one the probability 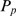 that the non-periodic gene is not needed, that means that our prediction is a false positive, is *γ*. For scenario three the probability that the non-periodic gene is actually non-periodic 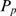 is 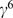 because there are six shortest paths this gene occurs on and the non-periodic gene is no longer needed as link between periodic genes if all of the six periodic genes at the end of the paths receive simultaneously a direct connection to another periodic gene. For scenario two one needs to distinguish two cases. 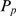 for the non-periodic genes closer to the SCC is 2*γ* because either the periodic genes receives a direct connection to another periodic gene or the second non-periodic gene receives a connection from a periodic gene. Both cases make the use of the first non-periodic gene on the shortest path redundant. 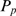 for the second non-periodic gene on the path is *γ* because this situation corresponds to scenario one. If *γ* would be known we could, for each non-periodic gene separately, estimate the probability that our prediction is a false positive. In general, for 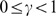 we obtain the ordering 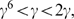
which makes scenario three (see Fig. 8) the most unlikely case to be a false positive prediction from a theoretical point of view. This corresponds to the fact that SWI5 is declared periodic by Cyclesbase [30] (see table 3). By this analysis we can assign the false positive probability *γ* to (STE12, RPH1, PIP2, SRD1) and 2*γ* to ADR1.

Next, we study the situation for false positive edges (edge removals). In the following we assume *δ* to be the probability of a false positive edge. First of all, we want to remark that the removal of edges can not create new paths but just destroy existing ones. This implies that there are two cases that need to be considered. First, the removal of an edge destroys the shortest path between two periodic genes and there exists no other path in the TRN that could connect these genes. In this situation additional edges need to be included (false negative edges need to exists) that would allow to create a new path. As mentioned above we will not consider such situations because such combinatorial events are increasingly unlikely (higher power in *γ* and/or *δ*). Second, removal of an edge makes an already existing path in the TRN a (new) shortest path connecting the two periodic genes. In the following we will assume that this is actually the case. Also for this situation we need to distinguish three scenarios (as described above). The probabilities for these three scenarios are 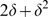, 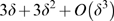 (for both genes regardless of their position on the path) and 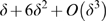. The term 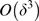 indicates that there are also terms of order 3 or higher in *δ* that influence the probabilities. For 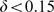 neglecting higher order terms we find the ordering (due to the non linearity of the equations there exist different regimes) 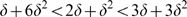
which corresponds to the ordering of the three scenarios for the false negative edges discussed above.

This gives the following combined ranking with the estimated false positive probability: 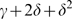 for (STE12, RPH1, SRD1), 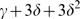 for (PIP2) and 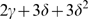 for ADR1.

### Visualization of expression profiles

**Figure 9:**
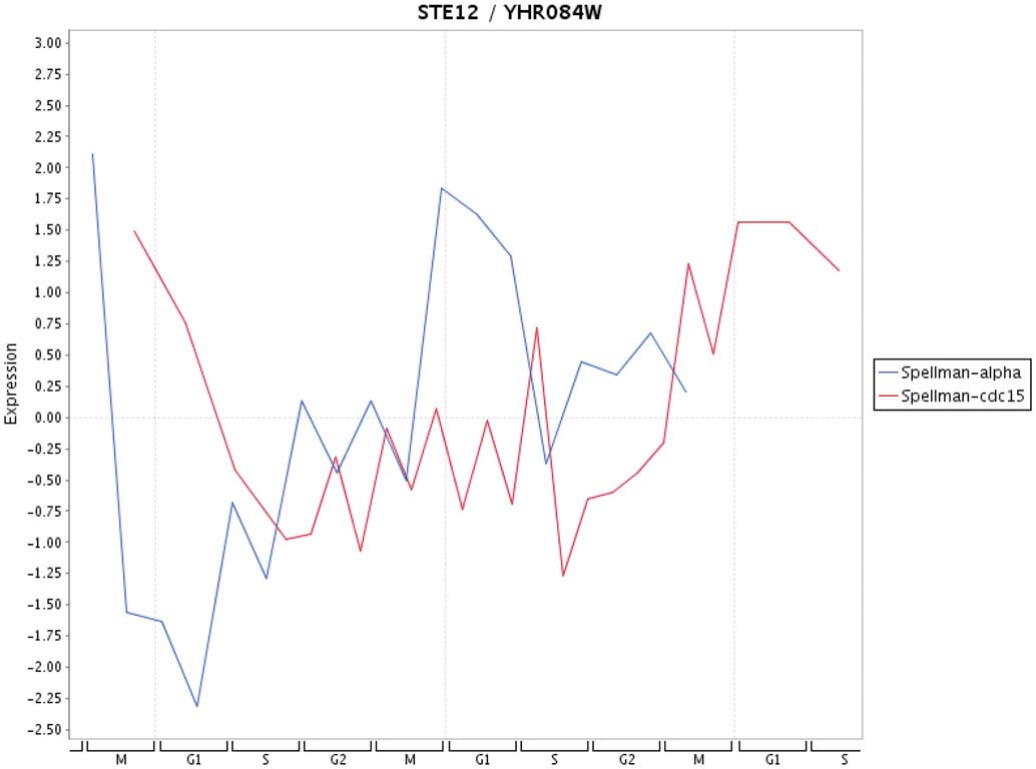
Expression profile for STE12 for time series data from Spellman et al. [32].

**Figure 10:**
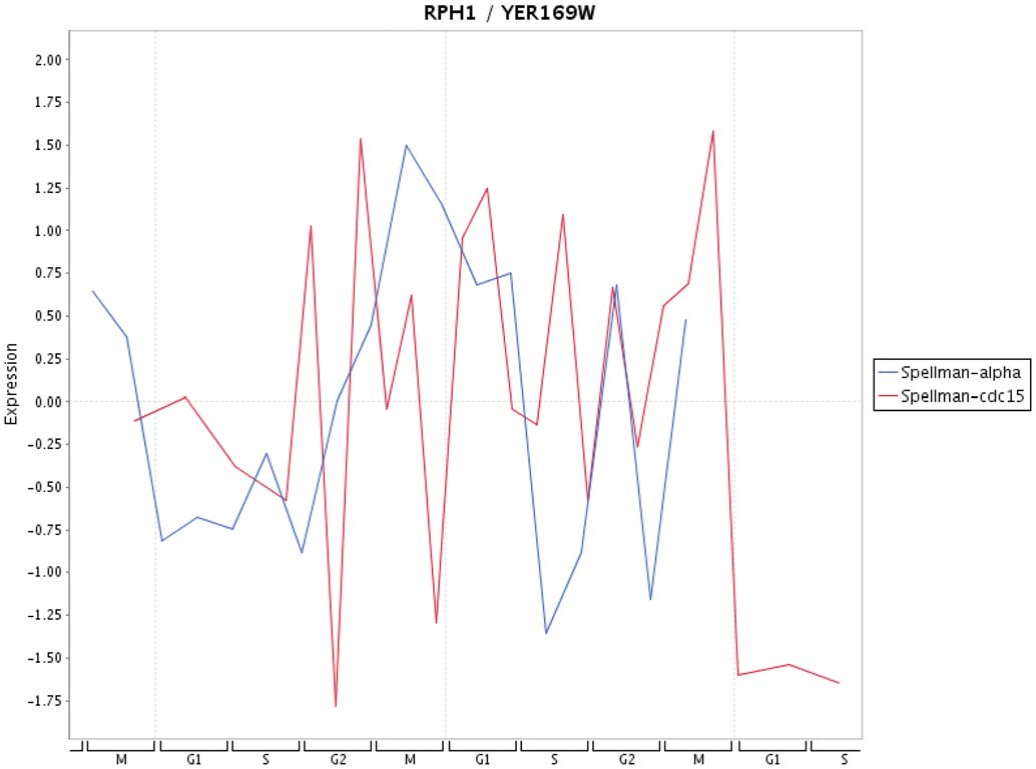
Expression profile for RPH1 for time series data from Spellman et al. [32].

**Figure 11:**
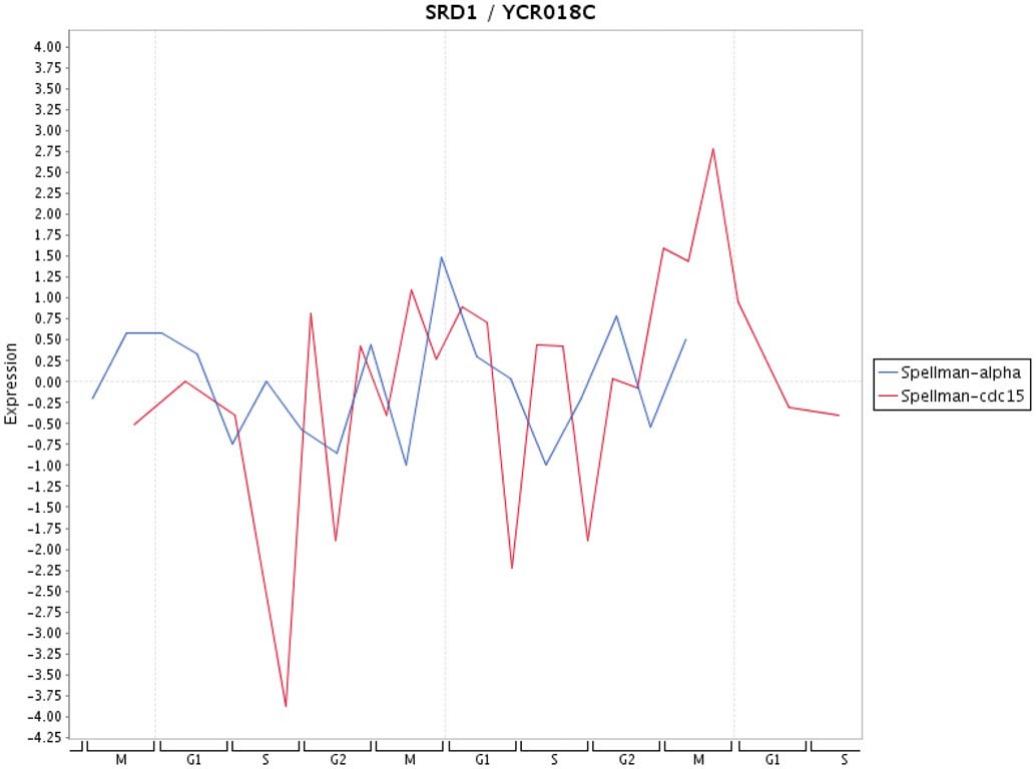
Expression profile for SRD1 for time series data from Spellman et al. [32].

**Figure 12:**
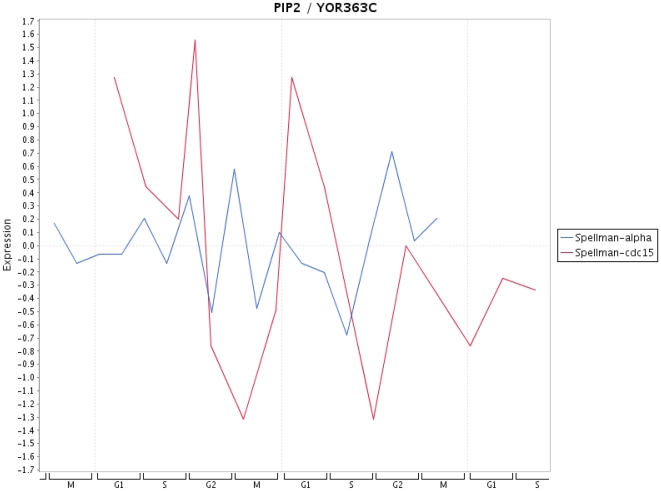
Expression profile for PIP2 for time series data from Spellman et al. [32].

**Figure 13:**
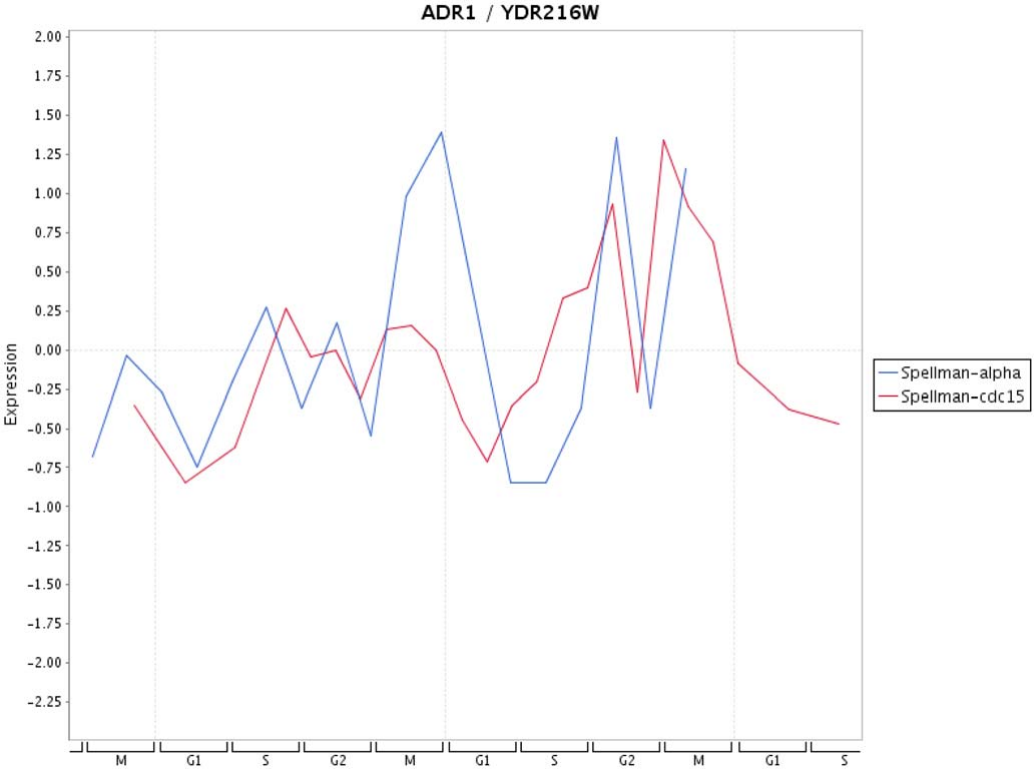
Expression profile for ADR1 for time series data from Spellman et al. [32].

Finally, in Fig. 9–13 we present a visualization of the expression profiles (obtained from Cyclebase) of the five genes predicted to be periodic. The time series used are from Spellman et al. [32]. In addition we provide in table 4 the p-values assigned by Cyclebase [30] for periodicity (second column) and for regulation (third column) of the five genes. The p-values for periodicity for PIP2 (Fig. 12) and ADR1 (Fig. 13) are below 0.05. Also, the p-values for regulation for STE12 and SRD1 are below 0.05. The reason why they are not declared as periodic is because their complementary p-value (either for regulation or periodicity) is much higher than 0.05. A possible reason for this is the high variability of the time series data with respect to different experiments. This variability makes it also very difficult to assign an unique peak time to these time series and, hence, for conventional methods based solely on the shape of a signal to clarify this situation. The only gene that has neither a low p-value for periodicity nor for regulation is RPH1 (Fig. 10). However, as one can see from Fig. 10 there are pronounced peaks occurring at certain phases of the cell cycle but these peaks are not precisely reproducible for different cycles and also experiments. This might be an indicator, if this gene is truly cell cycle regulated, of the redundancy of this gene meaning it is not involved in an unique signaling path but occurs on a parallel pathway that is not used during every cell cycle. This would provide a plausible explanation of the observed variability in the expression profile for different cell cycles as well as different experiments.

**Table 4:** P-values for periodicity and for regulation according to the evaluation of Cyclebase [30].

gene	p-value (periodic)	p-value (regulated)
STE12/YHR084W	5.79	0.0454
RPH1/YER169W	0.587	0.6726
SRD1/YCR018C	3.478	0.0042
PIP2/YOR363C	2.042E-7	0.6495
ADR1/YDR216W	0.003	0.4745

## Discussion

In this paper we presented a novel approach to predict genes causally involved in the cell cycle in *S. cerevisiae*. Our approach is based on the transcriptional regulatory network and a list of genes known to be periodic. No further data are used. Partitioning of the set of periodic genes in two groups according to a graph theoretical property leads to a hierarchy in the transcriptional regulatory network from the SCC to 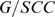 that allows to make predictions about the involvement of genes in the cell cycle. Based on our analysis we found five genes that are candidates to be periodically expressed. The estimated probability that theses genes are false positives is 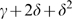 for (STE12, RPH1, SRD1), 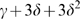 for (PIP2) and 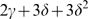 for ADR1. Here *γ* is the probability for a false negative edge and *δ* is the probability for a false positive edge. Generally, we want to remark that the property *cyclicity* of a network, used in this paper to define the SCC, has been already used previously to meaningfully separate molecular networks [33] but in the context to identify structural domains of proteins. Finally, we want to emphasize that our approach is not intented as alternative to existing methods to predict periodic genes but to complement such methods because we utilize different information.

From a theoretical point of view it would be interesting to study in a follow-up work the connection of our proposed method to a related framework based on Markov random fields [34]. Markov random fields have been used previously to predict the function of proteins by utilizing a protein network and information about functional categories of proteins for which such information is available [35]. This allows not only to predict a certain functional category for proteins but also to assess the confidence of this prediction. An interesting point would be to investigate the influence of the directedness of the network because Markov random fields are only defined for undirected networks whereas our approach utilizes the information provided by the directed edges. Also it would be interesting to study if our approach can be used to study undirected networks like the protein interaction network and under which assumptions.
